# Methionine aminopeptidase-2 blockade reduces chronic collagen-induced arthritis: potential role for angiogenesis inhibition

**DOI:** 10.1186/ar2340

**Published:** 2007-12-11

**Authors:** John Bainbridge, Leigh Madden, David Essex, Michael Binks, Rajneesh Malhotra, Ewa M Paleolog

**Affiliations:** 1 Kennedy Institute of Rheumatology, Faculty of Medicine, Imperial College London, 1, Aspenlea Road, London W6 8LH, UK; 2 Division of Surgery, Oncology, Reproductive Biology & Anaesthetics, Faculty of Medicine, Imperial College 1, Aspenlea Road, London, London W6 8LH, UK; 3 II CEDD, GSK Medicines Research Centre, Gunnels Wood Road, Stevenage SG1 2NY, UK

## Abstract

The enzyme methionine aminopeptidase-2 (MetAP-2) is thought to play an important function in human endothelial cell proliferation, and as such provides a valuable target in both inflammation and cancer. Rheumatoid arthritis (RA) is a chronic inflammatory disease associated with increased synovial vascularity, and hence is a potential therapeutic target for angiogenesis inhibitors. We examined the use of PPI-2458, a selective non-reversible inhibitor of MetAP-2, in disease models of RA, namely acute and chronic collagen-induced arthritis (CIA) in mice. Whilst acute CIA is a monophasic disease, CIA induced with murine collagen type II manifests as a chronic relapsing arthritis and mimics more closely the disease course of RA. Our study showed PPI-2458 was able to reduce clinical signs of arthritis in both acute and chronic CIA models. This reduction in arthritis was paralleled by decreased joint inflammation and destruction. Detailed mechanism of action studies demonstrated that PPI-2458 inhibited human endothelial cell proliferation and angiogenesis *in vitro*, without affecting production of inflammatory cytokines. Furthermore, we also investigated release of inflammatory cytokines and chemokines from human RA synovial cell cultures, and observed no effect of PPI-2458 on spontaneous expression of cytokines and chemokines, or indeed on the angiogenic molecule vascular endothelial growth factor (VEGF). These results highlight MetAP-2 as a good candidate for therapeutic intervention in RA.

## Introduction

Rheumatoid arthritis (RA) is a chronic inflammatory disease that is characterized by severe synovial inflammation, resulting in destruction of bone and cartilage. At the cellular level, early changes in the RA synovium include angiogenesis, inflammatory cell infiltration and synovial hyperplasia. Angiogenesis is defined as the process in which blood vessels form by the sprouting of pre-existing capillary plexuses [1]. It is a complex, highly regulated process found in physiological settings such as wound and fracture healing and during the female reproductive cycle. Otherwise, the vascular endothelium is maintained in a state of quiescence, which is the result of a tightly regulated system of opposing angiogenic regulators [2].

Angiogenesis is now also recognized to be a fundamental component of disease progression in RA [3]. Synovial blood vessel number has been found to correlate with hyperplasia, mononuclear cell infiltration and indices of joint tenderness [4]. Endothelial cells lining blood vessels within RA synovium have been shown to express cell cycle antigens such as PCNA (proliferating cell nuclear antigen) and Ki67 [5]. In the inflamed synovium, the normally quiescent angiogenic balance is disrupted in favour of angiogenesis by upregulated production of a variety of angiogenic stimulators, including vascular endothelial growth factor (VEGF) [6,7]. VEGF has been detected at higher levels in serum and synovial fluid from RA patients [8]. However, although blockade of VEGF has been proposed to be of potential therapeutic benefit in RA, emerging understanding of other potential functions of VEGF are making this molecule less attractive as a therapeutic target in the clinic. For example, reduced expression of VEGF has been reported to result in amyotrophic lateral sclerosis-like motor neurone degeneration in mice, suggesting a neuroprotective role for VEGF [9]. Thus, despite the considerable success of VEGF blockade in colorectal cancer, complications associated with anti-VEGF antibody, such as hypertension and gastrointestinal perforation, have prompted the search for other angiogenesis-associated targets.

Methionine aminopeptidase (MetAP)-2 is a metalloprotease that plays a key role in the removal of amino-terminal initiator methionines from nascent polypeptides [10]. The MetAP enzymes have been divided into two classes, with the MetAP-2 isoform being identified as the protein that irreversibly bound the angiostatic compounds fumagillin and its derivative AGM-1470/TNP-470 [11]. Previous reports have shown that fumagillin and AGM-1470/TNP-470 primarily function by inhibiting cell proliferation [11,12], and it has therefore been suggested that irreversible inhibition of MetAP-2 catalytic activity accounts for at least part of the anticancer action of such compounds [13-15]. Specifically, AGM-1470/TNP-470 has been reported to prevent the entry of endothelial cells into the G_1 _phase of the cell cycle [16]. A reversible MetAP-2 inhibitor was also reported to lead to endothelial cell cycle arrest and to exhibit efficacy in a range of murine tumour models [17]. Other MetAP-2 inhibitors have been described as inhibiting tumour growth in mice, and to induce accumulation in endothelial cells of the cyclin-dependent kinase inhibitor p21^WAF1/Cip1 ^[18].

It has been reported that fumagillin and other molecules that may act by inhibiting MetAP-2 reduce arthritis in animal models. In these models, disease is induced by immunizing susceptible strains of mice with collagen to generate an autoimmune response similar to that of RA, which includes inflammation at joints and joint destruction. Such collagen-induced arthritis (CIA) is in widespread use as a model of arthritis, and provides a useful tool with which to study the pathology of arthritis. We and others have shown that inhibition of angiogenesis, using either approaches targeting VEGF or other antiangiogenic strategies, ameliorates disease [19-25].

In the present study we utilized CIA to investigate further the therapeutic potential in RA of PPI-2458, an irreversible MetAP-2 inhibitor based on the fumagillin class of compounds. Our *in vivo *studies contained two models of arthritis with induction of CIA by bovine or murine collagen. When bovine collagen type II was used, the resultant disease was monophasic and characterized by severe synovial inflammation resulting in destruction of bone and cartilage. Disease severity was markedly reduced by PPI-2458. In addition to assessing the effects of PPI-2458 in acute CIA, we examined the effect of PPI-2458 in a model of chronic relapsing arthritis. We observed that PPI-2458, as well as reducing acute CIA, significantly decreased disease severity and joint destruction in chronic relapsing CIA. A potential mechanism of action of PPI-2458 *in vivo *was through reduction in angiogenesis, because *in vitro *studies showed endothelial proliferation and angiogenesis was inhibited by PPI-2458 whereas endothelial inflammatory responses were not affected. Similarly, release of tumour necrosis factor (TNF)-α, IL-6, and the chemokines IL-8, interferon-γ-inducible protein (IP)-10, macrophage inflammatory protein (MIP)-1α and monocyte chemoattractant protein (MCP)-1 by human RA synovial membrane cells remained unaffected. These findings provide support for the concept that blockade of MetAP-2 may be an effective approach to treatment of RA.

## Materials and methods

### Induction and monitoring of heterologous and homologous CIA

Bovine type II collagen was extracted from bovine articular cartilage and murine type II collagen was purified from mouse sternums. Purification was carried out as previously described [26].

For induction of acute heterologous CIA, 10-week-old male DBA/1 (H-2^q^) mice (Harlan UK Limited, Oxon, UK) received a single intradermal injection at the base of the tail of 100 μg bovine type II collagen emulsified in Freund's complete adjuvant, containing paraffin oil, and lyophilized *Mycobacterium tuberculosis *H37 Ra (Difco Becton Dickinson, Cowley, Oxford, UK). The first clinical signs of arthritis were deemed to be present when oedema and/or erythema involving any one of the four paws was observed. Onset of disease was observed from 2 weeks after collagen administration. For induction of chronic homologous CIA, 8-week-old DBA/1 (H-2^q^) male mice were used (Harlan UK Limited) and immunized following a protocol adapted from the previously described model [27], using an intradermal injection at the base of the tail of 100 μg murine type II collagen emulsified in Freund's complete adjuvant followed by a boost of 100 μg of murine type II collagen given at day 14. The first clinical signs of arthritis were assessed as for acute CIA, with onset of disease occurring from 2 weeks after murine type II collagen boost.

In both models mice were monitored daily and each limb was assigned a clinical score as follows: 0, normal paws and no clinical features of inflammation; 1, slight oedema or erythema; 2, pervading oedema/erythema involving the entire paw; and 3, pronounced oedema and erythema leading to incapacitated limb mobility. All mice used in this study were housed in individual ventilated cages over a 20-hour light/dark cycle and were fed standard laboratory chow and water *ad libitum*. Animal work was conducted under the Home Office project licence PPL no: 70/5446 'Pathogenesis and therapy for RA' under the operatives of the Animals (Scientific Procedures) Act 1986, which followed the principles of the Helsinki Declaration.

**Figure 1:**
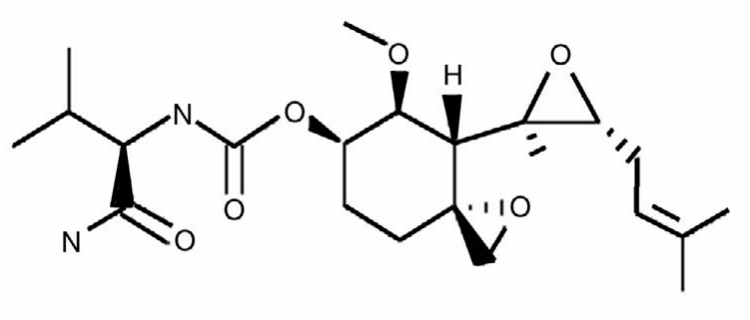
Structure of PPI-2458.

In both models, animals were treated with PPI-2458 [28], a member of the fumagillin class of antiantigenic compounds. Fumagillin and its analogues are reported to be potent, irreversible and selective inhibitors of MetAP-2 [29]. PPI-2458 was synthesized by the method covered in the Praecis Pharmaceuticals patent WO 02/42295 2002, priority US 704251 2000. The structure of the molecule is shown in Figure 1.

Treatments were administered from the first day of visible arthritis. Mice with acute CIA were treated daily intraperitoneally with vehicle control (saline) or PPI-2458 at 0.5 mg/kg, 1.5 mg/kg, or 5 mg/kg for 10 days. For chronic CIA, treatments were every other day intraperitoneally as follows: vehicle (sterile 0.85% physiological saline), murine soluble TNF receptor (murine p75 receptor dimer fused to murine IgG_2a_; soluble TNF receptor [sTNF-R]II) at 5 mg/kg, PPI-2458 at 1.5 mg/kg and indomethacin (Sigma Aldrich Co., Poole, UK) at 2.5 mg/kg. Two chronic CIA experiments were performed, with the first terminated 4 weeks after onset of symptoms (day 29 of arthritis) and the second terminated 10 weeks after onset of symptoms (day 71 of arthritis).

### Histopathology

At the end of the experiments the hind feet of mice were excised after euthanasia and fixed in 10% buffered formalin solution. Fixed specimens were decalcified (Rapid-Cal™; BBC Biochemical, Dallas, TX, USA) and embedded in paraffin wax. Serial sections of 5 μm thickness were obtained, dewaxed, and stained with haematoxylin and eosin. The stained sections were scored microscopically for changes to joint architecture by an observer who was blinded to the study groups. Paws were assigned one of four grades: normal; mild synovitis with some loss of cartilage; moderate with more extensive synovial hyperplasia, destruction of cartilage and some bone erosions caused by an invasive pannus front; and finally severe, namely to have complete destruction of the joint architecture. Data were analysed by χ^2 ^test comparison of the number of animals exhibiting normal, mild, moderate and severe changes versus untreated mice.

### Endothelial cell studies

Human umbilical cords were collected from Chelsea and Westminster Hospital (London, UK) in accordance with the guidelines of the Riverside Research Ethics Committee (RREC 2948). Human umbilical vein endothelial cells (HUVEC) were isolated by digestion of umbilical cord veins in 0.025 mg/ml collagenase A (Roche Diagnostics, Mannheim, Germany) [30]. Cells were maintained in RPMI-1640 (Cambrex, Berkshire, UK) containing 10% (vol/vol) foetal calf serum (FCS; Biowest, Nuaillé, France), 10% (vol/vol) new born calf serum (Gibco, Paisley, UK), 5 U/ml heparin (CP Pharmaceuticals, Wrexham, UK), and 15 μg/ml endothelial cell growth supplement (Sigma Aldrich Co.) at 37°C, in an atmosphere of 5% carbon dioxide and 95% air. Endothelial cells between second and third passage were used for all experiments.

A modified assay for mitochondrial activity was used to assess cell viability. Wells were treated with fumagillin (GSK, Stevenage, UK) at a concentration of 10 nmol/l or PPI-2458 (0.01 to 10 nmol/l). Following treatment as above, 0.5 mg/ml MTT (3-[4,5-dimethyl-2-yl]-2,5-diphenyltetrazolium; Sigma Aldrich Co.) was added to the cells overnight. Cells were lysed using 10% SDS containing 0.001 mol/l HCl and absorption was measured after 24 hours at 620 nm using a using a Multiskan Ascent Scanner plate reader (Thermo Labsystems, Basingstoke, UK).

To determine the effect of PPI-2458 on proliferation, HUVEC were seeded at 3 × 10^3 ^cells per 30 mm^2 ^wells (25% confluence), in RPMI-1640 containing 10% FCS, 10% new born calf serum, 5 U/ml heparin and 15 μg/ml endothelial cell growth supplement for 24 hours. Medium was then changed to different conditions (as described for each experiment) for 24 hours and cells were pulsed with 0.5 μCi ^3^H-thymidine (GE Healthcare, Chalfont St Giles, UK) for a further 24 hours. At the end of the experiment, cells were subjected to three freeze-thaw cycles before being analyzed for ^3^H-thymidine incorporation using a cell harvester (Micro-96-Harvester; Skatron Instruments, Corston, UK). Human recombinant VEGF and human recombinant fibroblast growth factor (FGF)-2 were obtained from R&D Systems (Abingdon, UK).

To analyze the effect of PPI-2458 on angiogenesis, a commercially available angiogenesis kit was used (AngioKit; TCS Cell Works, Buckingham, UK). Cultures spontaneously developed a network of capillary-like tubules after 11 days at 37°C in 5% carbon dioxide. Wells were treated on day 0 with VEGF (2 ng/ml) or FGF-2 (50 ng/ml), in the absence or presence of either fumagillin (10 nmol/l) or PPI-2458 (1 to 10 nmol/l). Culture medium was replenished after 4, 7, and 9 days, in accordance with the manufacturer's instructions. On day 11, the medium was aspirated and the plates were fixed at room temperature in 70% ethanol that had been stored at -20°C. Expression of CD31 was visualised by staining with mouse antihuman CD31 antibody (TCS Cell Works) for 60 minutes at 37°C, followed by goat antimouse IgG alkaline phosphatase conjugate for 10 minutes at room temperature. CD31 enzyme-linked immunosorbent assay (ELISA) substrate was prepared by dissolving p-nitrophenol phosphate in Tris buffer and was added in accordance with the manufacturer's instructions. The plate was then read against blank wells at 405 nm using a plate reader Multiskan Ascent plate reader (Thermo Labsystems). Subsequently, an insoluble substrate prepared from BCIP/NBT (5-bromo-4-chloro-3-indolyl phosphate/nitro blue tetrazolium; TCS Cell Works) was added. Once the substrate was filtered, 0.5 ml was added per well and incubated at 37°C until tubules developed a dark colour (5 to 15 minutes). Wells were then washed three times with distilled water and air dried before microscopic analysis using a BH2 microscope (Olympus Optical, London, UK) linked to a KY-F55BE video camera (JVC, London, UK). Digital images were processed using AnalySIS software (Soft Imaging Software, Munster, Germany).

To determine the effect of PPI-2458 on cytokine release, HUVEC were plated at 3 × 10^4 ^in 75 mm^2 ^wells and stimulated for 24 hours with either IL-1β (10 ng/ml; BioSource, UK), TNF-α (10 ng/ml; R&D Systems), or lipopolysaccharide (LPS; 500 ng/ml; from *Escherichia coli *0111:B4 cell culture tested; Sigma Aldrich Co.) in RPMI-1640 containing 10% FCS. Fumagillin (10 nmol/l) and PPI-2458 (0.01 to 10 nmol/l) were added where indicated, or 0.001% dimethyl sulfoxide as a vehicle control. Release of MCP-1 was measured by ELISA. Briefly, mouse monoclonal antihuman C-C chemokine ligand-2/MCP-1 IgG_2b _(R&D Systems) was coated onto polystyrene microtitre plates (Nunc-Immunoplate II; BRL, Uxbridge, Middlesex, UK) at a concentration of 2 μg/ml overnight at 4°C. After blocking with 2% bovine serum albumin in phosphate-buffered saline, samples or MCP-1 standard (human recombinant MCP-1; R&D Systems) were added overnight at 4°C. Bound MCP-1 was detected using biotinylated antihuman MCP-1 IgG (R&D Systems) at a concentration of 25 ng/ml, followed by streptavadin-horseradish peroxidase (Amersham Life Sciences Ltd, Little Chalfont, UK) and 3,3',5,5'-tetramethylbenzidine peroxidase substrate (Kirkegaard and Perry Laboratories, Gaithersburg, MA, USA). The colour reaction was then stopped with 2 mol/l H_2_SO_4 _and absorbance was read at 450 nm on a Multiskan Ascent Scanner plate reader (Thermo Labsystems).

### RA synovial cell culture studies

Synovial tissue was harvested from patients with a diagnosis of RA, made by rheumatologists who referred patients to Mount Vernon Hospital (Northwood, Middlesex, UK) for hand surgery (Local Ethics Research Committee EC2003-64). Tissue was washed in sterile RPMI-1640 and cut into small pieces using sterile scissors and surgical forceps. The resultant tissue was then digested in Dulbecco's modified Eagle's medium (Lonza, Wokingham, UK) containing 10% FCS, 1 g/l collagenase (Roche Diagnostics, Mannheim, Germany) and 0.15 g/l DNAase (type IV from bovine pancreas; Sigma Aldrich Co.) for up to 1 hour at 37°C. The disaggregated cells were filtered through a mesh to remove debris. Cell viability was assessed using trypan blue exclusion. Viable cells were cultured at 10^6^/ml in a volume of 125 μl in Dulbecco's modified Eagle's medium containing 10% FCS at 37°C in 75 mm^2 ^wells in the absence or presence of either Fumagillin (10 nmol/l), PPI-2458 (0.1 to 10 nmol/l), or 0.001% dimethyl sulfoxide as vehicle control. After 24 hours, supernatants were removed, and cytokine production was assayed using a Beadlyte Human Multiplex Cytokine Detection System (Upstate Biotechnology, Lake Placid, NY, USA). Samples or standards were incubated with the cytokine capture bead suspension array, in accordance with the manufacturers' instructions. Beads were washed using a vacuum manifold and biotinylated reporter antibodies were added, followed by streptavidin-phycoerythrin. The median fluorescence intensity of 50 beads per cytokine was read using a Luminex 100™ (Luminex, Austin, TX, USA) and analysed using STarStation analysis software (Applied Cytometry Systems, Sheffield, UK).

### Statistical analysis

Data were analysed using GraphPad Prism 4.03 (GraphPad Software, San Diego, CA, USA). For CIA experiments, analysis of different treatments was carried out with a two-way analysis of variance. Histology data were analysed by χ^2 ^test. A one-way analysis of variance with Bonferroni post-test for multiple comparisons was used to compare different groups of data for *in vitro *studies. *P *values less than 0.05 were considered to be statistically significant.

## Results

### Therapeutic effect of PPI-2458 in acute and chronic relapsing CIA

The acute and chronic CIA murine models were used to assess the effect of the MetAP-2 inhibitor PPI-2458 as a potential therapeutic agent. In acute CIA, the incidence of arthritis in each cage reached at least 80%. The earliest signs of disease were observed on day 14 after immunization, with a median day of arthritis onset of day 28 after immunization. In chronic CIA there were clinical signs of arthritis in at least 70% of animals per cage. Animals became arthritic from day 28 after primary immunisation (14 days after murine collagen type II boost), with a median day of arthritis onset of day 49 after immunization.

**Figure 2:**
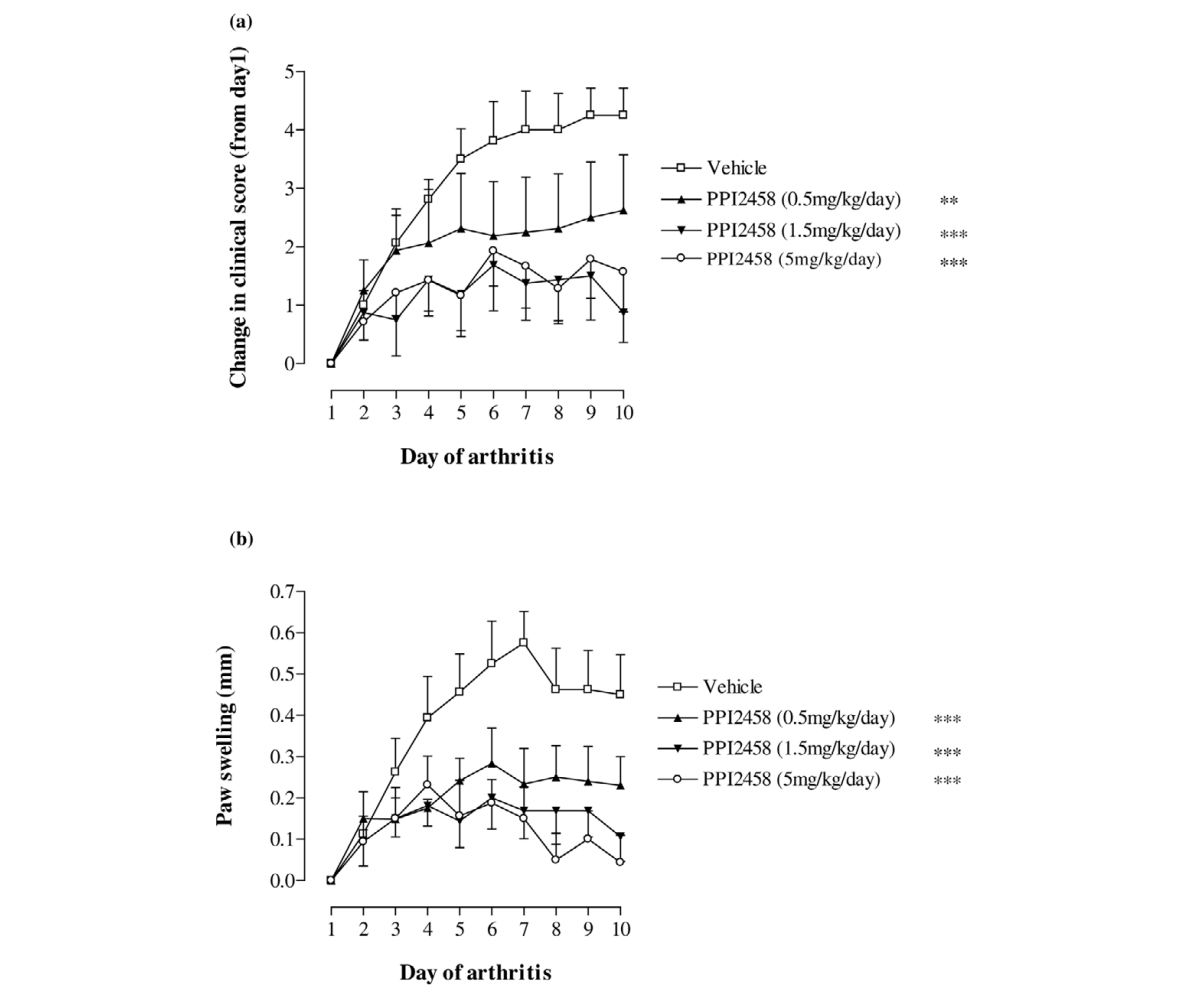
PPI-2458 reduces acute CIA. Following onset of arthritis induced by bovine type II collagen, mice were treated with PPI-2458 (0.5, 1.5, or 5 mg/kg per day) or vehicle alone (*n *= 8 per group). **(a) **Change in clinical score for each animal from day 1 of arthritis and **(b) **paw swelling were assessed over a 10-day period. Data are expressed mean ± standard error. Statistical analysis was carried out using a two-way analysis of variance versus vehicle-treated mice: ** *P *< 0.01, ***P < 0.001. CIA, collagen-induced arthritis.

In acute CIA, the mice treated with vehicle control developed the typical signs of acute CIA, with a rapidly progressing monophasic disease. Both clinical score (Figure 2a) and paw swelling (Figure 2b) increased with time until day 8 after onset, when a plateau was apparent. Treatment with PPI-2458 at all three doses (0.5, 1.5 and 5 mg/kg per day) showed a significant arrest in the development of arthritis, which was highly statistically significant. Treatment with PPI-2458 at 0.5 mg/kg per day was slightly less effective than treatment with either 1.5 mg/kg per day PPI-2458 (*P *< 0.05 versus 0.5 mg/kg per day) or 5 mg/kg per day PPI-2458 (*P *< 0.05 versus 0.5 mg/kg per day).

**Figure 3:**
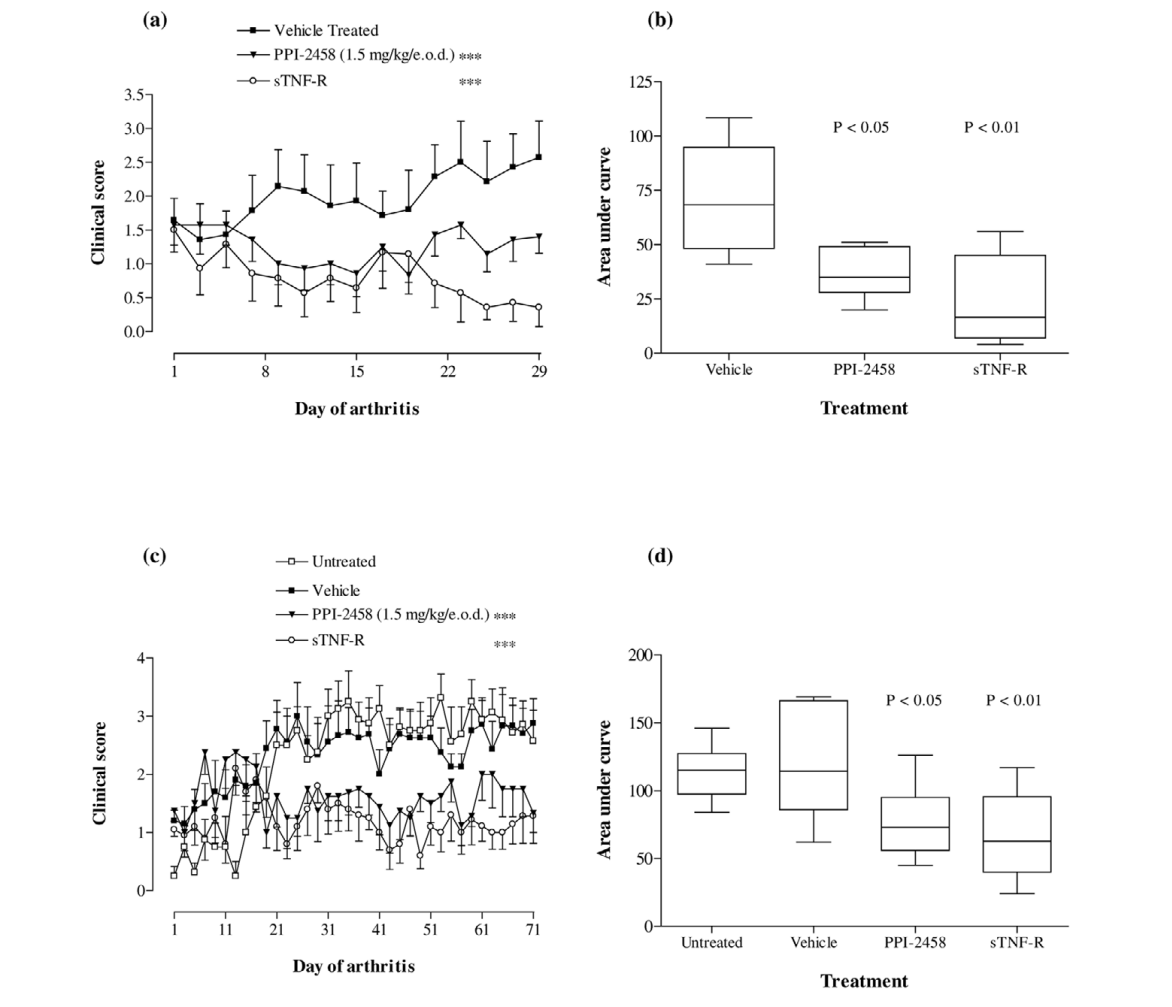
PPI-2458 reduces chronic CIA. Following onset of arthritis induced by murine type II collagen, mice were treated with either PPI-2458 (1.5 mg/kg every other day), soluble tumour necrosis factor receptor (sTNF-R)II (5 mg/kg every other day) or vehicle alone, or were left untreated (as indicated for the relevant experiments). **(a) **Clinical score was assessed over 4 weeks. Data are expressed mean ± standard error. Statistical analysis was carried out using a two-way analysis of variance versus vehicle-treated mice: ***P < 0.001. Each treatment group contained at least six mice. **(b) **Area under curve analysis, with data represented as box-and-whiskers plots. Statistical analysis was carried out using a one-way analysis of variance versus vehicle-treated mice: *P < 0.05, **P < 0.01. **(c) **Clinical score was assessed over 10 weeks. Data are expressed mean ± standard error. Statistical analysis was carried out using a two-way ANOVA versus vehicle-treated mice: ***P < 0.001. Each treatment group contained at least eight mice. **(d) **Area under curve analysis, with data represented as box-and-whiskers plots. Statistical analysis was carried out using a one-way analysis of variance versus vehicle-treated mice: *P < 0.05, **P < 0.01.

Chronic CIA manifested differently to the bovine type II collagen induced acute CIA, and was primarily observed as increased reddening of the paw with individual joints affected and progressive increase in the number of involved joints, with a milder course of disease (Figure 3). In the first instance, the clinical score was measured over 28 days after onset. In mice treated with vehicle (saline), the mean clinical score exhibited a gradual increase with time (Figure 3a). A comparable pattern was seen in a subsequent experiment, in which mice were monitored for up to 70 days after onset (Figure 3c). Treatment with either PPI-2458 or with murine sTNF-RII (used as a control) for comparison significantly (*P *< 0.001 versus vehicle-treated animals) reduced arthritis severity in both experiments. For example, on day 71 of chronic arthritis, untreated mice exhibited a mean (± standard error) clinical score of 2.57 ± 0.53, as compared with 1.33 ± 0.33 and 1.28 ± 0.47 for mice treated with PPI-2458 and sTNF-RII, respectively. In the same experiment, vehicle-treated mice exhibited a clinical score of 2.87 ± 0.53.

Area under the curve analysis demonstrated a significant reduction in disease severity in both experiments, relative to vehicle-treated mice (Figures 3b,d). Such analysis allowed for additional comparison of disease severity in this model, in which remission and relapse occurred on different days for individual animals. In the experiment terminated on day 29 of arthritis, the median (interquartile range) area under the curve for vehicle-treated mice was 68.25 (range 48.25 to 94.75), as compared with 16.50 (7.00 to 45.00) and 35.00 (28.00 to 49.00) for sTNF-RII and PPI-2458 treated animals, respectively. Similarly, in the experiment terminated on day 71 of arthritis, the median (interquartile range) area under the curve for untreated and vehicle-treated mice was 115.00 (97.50 to 127.30) and 114.30 (86.00 to 166.30), as compared with 62.75 (40.00 to 95.50) and 73.00 (56.00 to 95.00) for sTNF-RII and PPI-2458 treated animals, respectively.

**Figure 4:**
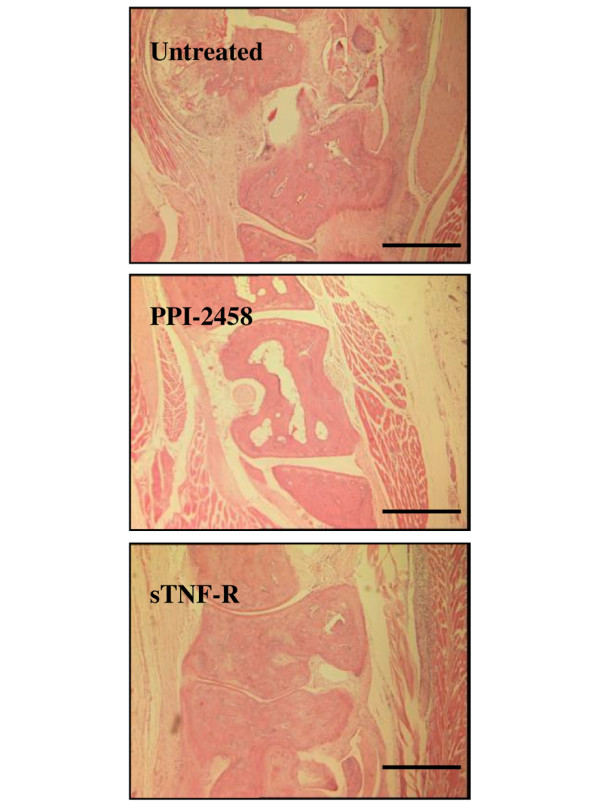
PPI-2458 reduces joint destruction in chronic collagen-induced arthritis. Following onset of arthritis, mice were treated with either PPI-2458 (1.5 mg/kg every other day), soluble tumour necrosis factor receptor (sTNF-R)II (5 mg/kg every other day), or vehicle alone for 28 days. Hematoxylin and eosin sections were scored in a blinded manner for pannus formation, synovitis, bone and cartilage erosion. Representative images, with bar equivalent to 20 μm, are illustrated.

Analysis of chronic CIA paw histology revealed increased cellular infiltrate. This was accompanied by bone loss and evidence of new woven bone formation. Compared with untreated mice, both PPI-2458-treated mice (*P *= 0.0015 versus untreated mice, by χ^2 ^test) and sTNF-RII-treated mice (*P *< 0.001) exhibited significant improvement in joint histology (Figure 4 and Table 1) on day 29 of arthritis. The percentages of animals exhibiting normal joint histology or only mild synovitis were 48% and 75%, respectively, as compared with just 11% for untreated animals. In the same experiment, indomethacin (2.5 mg/kg every other day) treatment significantly reduced clinical score (*P *< 0.001 versus untreated mice; data not shown), but was without significant effect on joint destruction (*P *= 0.1074; data not shown), which is in agreement with previously published data for this arthritis model [27]. Similarly, 39% of untreated animals killed on day 71 of arthritis exhibited normal joint histology or only mild synovitis, as compared with 50% for sTNF-RII-treated mice (*P *= 0.023 versus untreated mice) and 78% for PPI-2458-treated mice (*P *= 0.009; data not shown).

**Table 1:** PPI-2458 reduces joint destruction in chronic collagen-induced arthritis

Arthritic changes	Treatment		
	
	Untreated	sTNF-RII	PPI-2458
Normal	0 (0%)	3 (19%)	0 (0%)
Mild	2 (11%)	9 (56%)	13 (56%)
Moderate	12 (67%)	4 (25%)	10 (44%)
Severe	4 (22%)	0 (0%)	0 (0%)
Significance	-	*P *< 0.001	*P *< 0.01

Following onset of arthritis, mice were treated with either PPI-2458 (1.5 mg/kg every other day), soluble tumour necrosis factor receptor (sTNF-R)II (5 mg/kg every other day), or vehicle alone for 28 days. Hematoxylin and eosin sections were scored in a blinded manner for pannus formation, synovitis, bone and cartilage erosion. Paws were assigned one of four grades: normal; mild synovitis with some loss of cartilage; moderate with more extensive synovial hyperplasia, destruction of cartilage and some bone erosions caused by an invasive pannus front; and finally severe, judged to have complete destruction of the joint architecture. Results are the number of paws exhibiting normal, mild, moderate and severe changes, with percentage in parentheses. Statistical analysis of raw data was carried out by χ^2 ^test versus untreated mice.

### Endothelial proliferation and angiogenesis are inhibited by PPI-2458

**Figure 5:**
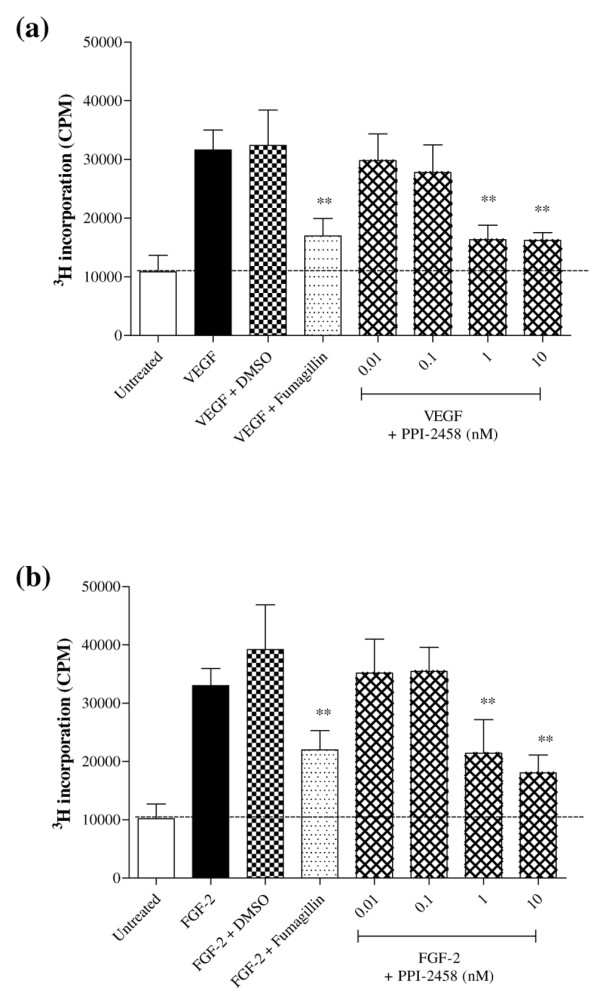
PPI-2458 reduces proliferation of HUVEC. Human umbilical vein endothelial cells (HUVEC) were plated at 3 × 10^3 ^in 30 mm^2 ^wells and stimulated with either **(a) **vascular endothelial growth factor (VEGF; 10 ng/ml) or **(b) **fibroblast growth factor (FGF)-2 (50 ng/ml) in 10% foetal calf serum, or were left untreated (serum-free medium). Fumagillin (10 nmol/l) and PPI-2458 (0.01 to 10 nmol/l) or 0.001% dimethyl sulfoxide as a vehicle control were added where indicated for 24 hours. Cells were pulsed with 10 μCi per well ^3^H-thymidine for a further 24 hours. Data are expressed as means ± standard deviation, representative of three experiments, and were analysed by one-way analysis of variance versus response in the absence of inhibitor: **P < 0.01.

**Figure 6:**
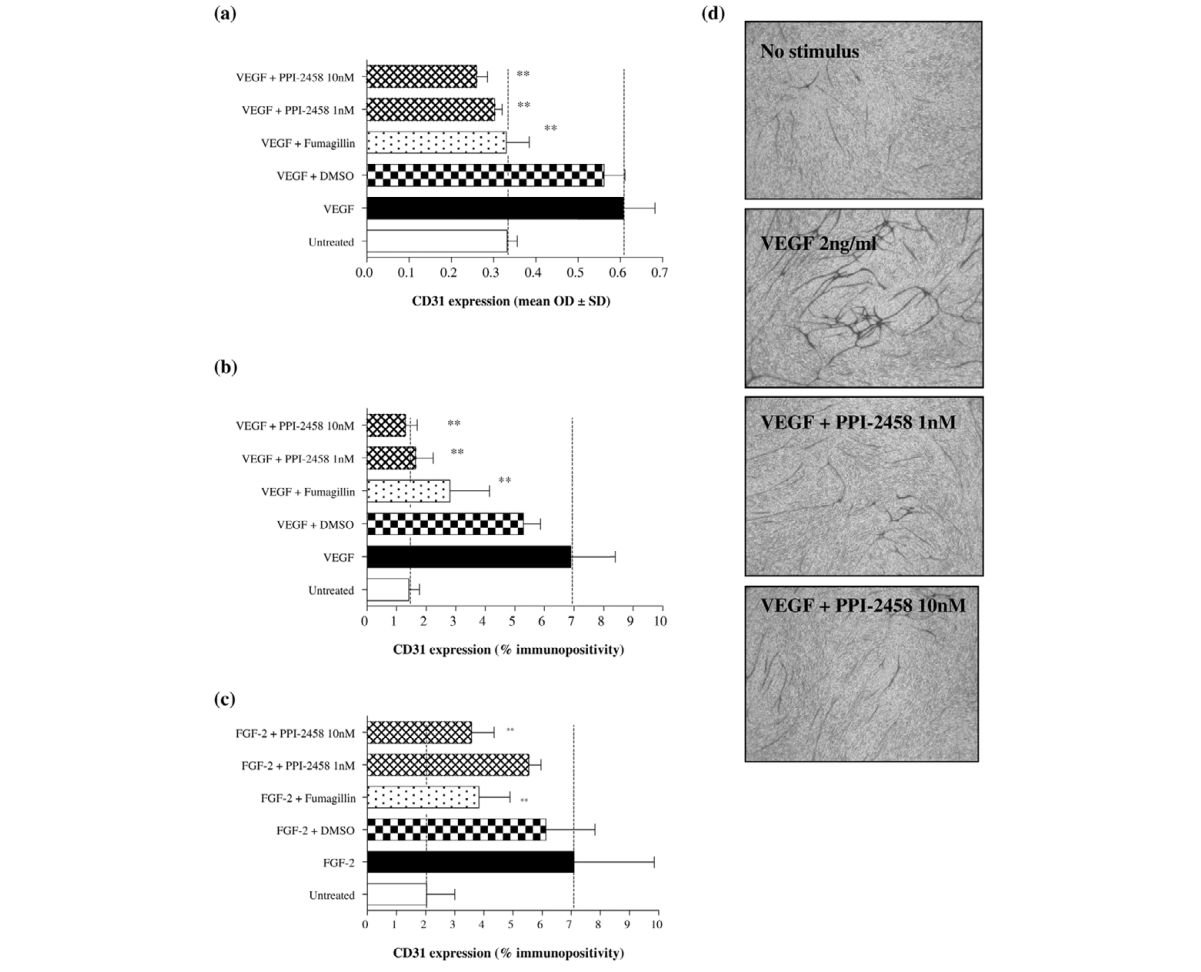
PPI-2458 reduces VEGF and FGF-2 mediated angiogenesis. Angiogenesis in response to either **(a,b,d) **2 ng/ml vascular endothelial growth factor (VEGF) or **(c) **50 ng/ml fibroblast growth factor (FGF)-2 was assessed after 11 days. Fumagillin (10 nmol/l) and PPI-2458 (1 nmol/l or 10 nmol/l), or 0.001% dimethyl sulfoxide as a vehicle control were added where indicated. CD31 expression was measured using alkaline phosphatase-mediated conversion of (panel a) p-nitrophenol phosphate followed by colorimetric assay (panels b and c) BCIP/NBT (5-bromo-4-chloro-3-indolyl phosphate/nitro blue tetrazolium) followed by image analysis. Data are expressed as means ± standatd deviation, representative of three experiments, and were analysed by one-way analysis of variance versus response in the absence of inhibitor: **P < 0.01. Included in panel d are representative images showing morphology of the formed tubes stained for CD31 at day 11 (objective magnification 40×).

Treatment of HUVEC with PPI-2458 significantly inhibited proliferation induced by either VEGF (Figure 5a) or FGF-2 (Figure 5b), assessed as incorporation of ^3^H-thymidine. PPI-2458 at 1 nmol/l was as effective as fumagillin at a dose of 10 nmol/l. Furthermore, the effect of PPI-2458 on angiogenesis was assessed. Both VEGF and FGF-2 increased angiogenesis, measured as CD31 expression either using p-nitrophenol phosphate or as CD31 immuno-positive area (Figure 6). PPI-2458 at 1 nmol/l and 10 nmol/l significantly reduced angiogenesis induced both by VEGF and FGF-2 (Figure 6a–c). Visualization of the endothelial network confirmed these results, with tubule formation being far less extensive following PPI-2458 and fumagillin treatment compared with untreated controls (Figure 6d).

**Figure 7:**
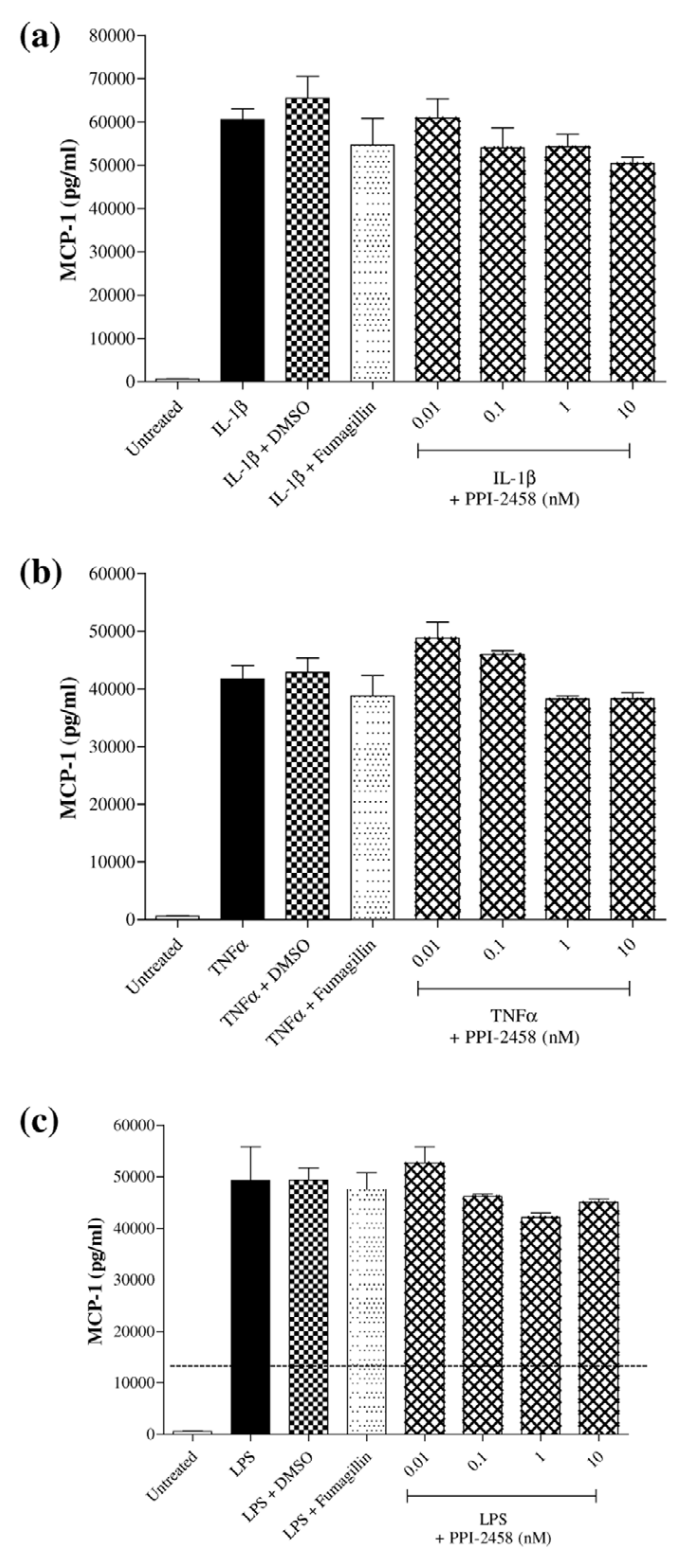
PPI-2458 does not affect HUVEC inflammatory responses. Human umbilical vein endothelial cells (HUVEC) were plated at 3 × 10^4 ^in 75 mm^2 ^wells and stimulated for 24 hours with either **(a) **IL-1β (10 ng/ml), **(b) **tumour necrosis factor (TNF)-α (10 ng/ml), or **(c) **lipopolysaccharide (LPS; 500 ng/ml). Fumagillin (10 nmol/l) and PPI-2458 (0.01 to 10 nmol/l), or 0.001% dimethyl sulfoxide as a vehicle control were added where indicated. Release of monocyte chemoattractant protein (MCP)-1 was measured by enzyme-linked immunosorbent assay (ELISA). MCP-1 was measured by ELISA. Data are expressed as mean ± standard deviation, representative of at least three experiments.

In contrast, incubation of confluent HUVEC with PPI-2458 (0.01 to 10 nmol/l) was without effect on release of cytokines in response to inflammatory stimuli (IL-1β, TNF-α, or LPS). This is illustrated in Figure 7 for production of MCP-1. Comparable results were obtained when release of IL-6 and IL-8 were measured (data not shown).

### PPI-2458 is without effect on RA synovial cell responses

**Figure 8:**
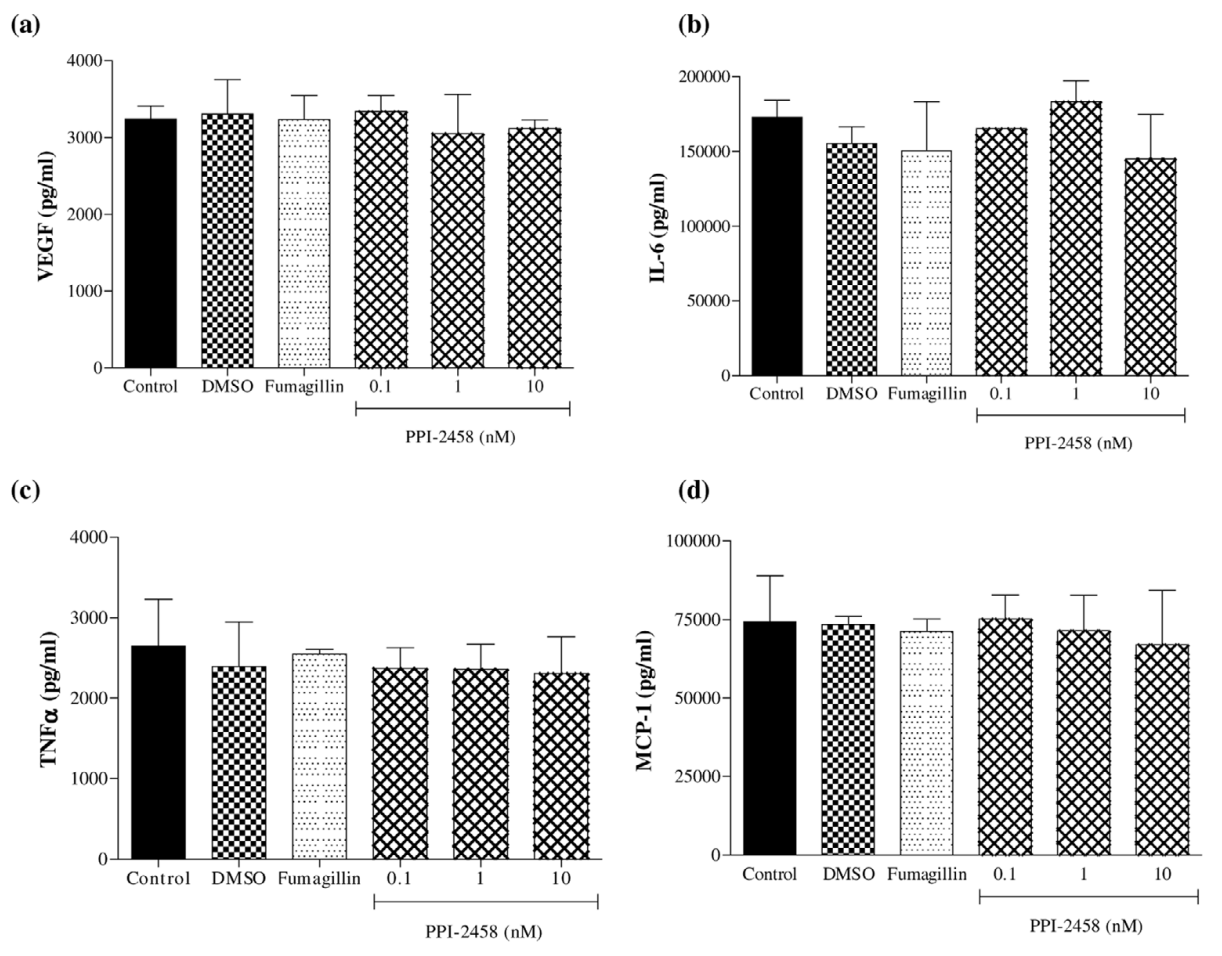
PPI-2458 does not affect RA synovial cell inflammatory responses. Rheumatoid arthritis (RA) synovial cells were plated at 5 × 10^5 ^in 75 mm^2 ^wells and incubated for 24 hours with either fumagillin (10 nmol/l), PPI-2458 (0.01 to 10 nmol/l), or 0.001% dimethyl sulfoxide as a vehicle control. Release of **(a) **vascular endothelial growth factor (VEGF), **(b) **IL-6, **(c) **transforming growth factor (TNF)-α and **(d) **monocyte chemoattractant protein (MCP)-1 were measured using enzyme-linked immunosorbent assay.

To determine whether PPI-2458 might exert effects on cells in RA synovium other than endothelial cells, RA synovial tissue was enzymatically dissociated, and release of cytokines and chemokines in the presence of increasing concentrations of either fumagillin or PPI-2458 was measured. Spontaneous productions of VEGF, IL-6, TNF-α and the chemokine MCP-1 were not affected at doses of PPI-2458 up to 10 nmol/l (Figure 8a–d). Comparable data were obtained when release of IP-10, MIP-1α and IL-8 were assessed (data not shown). Cell viability was also not affected in these studies (data not shown).

## Discussion

Endothelial cell proliferation is important in RA pathogenesis, providing the foundation of leucocyte invasion, synovial inflammation, pannus formation and bone destruction. Angiogenesis has been implicated in the pathophysiology of RA. Furthermore, molecules with MetAP-2 inhibitory activity such as TNP-470 and fumagillin can suppress disease in animal models, possibly via inhibition of angiogenesis *in vivo *[31-34]. Previous studies have reported that established CIA, induced in rats by chick collagen, was significantly reduced by treatment with the MetAP-2 inhibitor AGM-470 [31-33]. AGM-1470 reduced joint destruction and vascularity, without affecting T-cell function [34]. Similarly, in the KRN (K/BxN)/nonobese diabetic model of spontaneous murine arthritis, TNP-470 suppressed disease when given early during the course of arthritis [35]. In severe combined immunodeficient mice implanted with human RA synovial tissue, the number of synovial lining cells and blood vessel counts was markedly reduced by TNP-470 compared with untreated animals. In parallel, proliferating endothelial cells in the implanted synovial tissue were also reduced by TNP-470 treatment [36].

In the present study, we utilized the acute CIA model in DBA/1 mice, which was used to demonstrate efficacy of anti-TNF-α antibody and which was the basis for the widespread clinical use of TNF-α inhibitors for treatment of patients with RA [37-40]. Acute murine CIA is thus an excellent predictive model in which to study new therapeutic approaches for RA. In parallel, we used a model of chronic CIA. Immunization of mice with murine rather than bovine collagen leads to disease that more closely resembles RA, supporting its use as a tool for testing new therapies [27].

We report that PPI-2458, an inhibitor of MetAP-2, not only inhibits acute CIA model but also has profound effect on the pathology of chronic CIA. Although heterologous CIA response is well known to involve predominantly a largely neutrophilic infiltrate, the success of PPI-2458 in ameliorating disease progression provides evidence of the crucial role of the vasculature in this model. Although all doses moderated arthritis, the most pronounced response was at 1.5 mg/kg per day, indicating that this was the optimal dose in this model. Our results are also supported by a study using an alternative model of arthritis, in which PPI-2458 was able to reduce paw swelling in Lewis rats with peptidoglycan-polysaccharide induced arthritis [28,41].

Whilst heterologous (bovine) CIA provides a tried and tested mechanism for assessing therapeutics such as TNF-α inhibitors [37-40], previous studies have shown that autologous type II collagen can be used to produce a chronic relapsing model with even greater relevance to RA [27,42]. The arthritis in these animals fluctuates between disease and improvement, involving with time an increasing number of joints, with remissions becoming less frequent. Histological findings indicate that the synovial infiltration comprises mostly mononuclear cells and that the synovium is characterized by an abundance of blood vessels, as is the case for RA. The usefulness of chronic CIA as a tool for testing new therapies is substantiated by the observation that chronic CIA can distinguish between anti-inflammatory and disease-modifying drugs. Indomethacin, a nonsteroidal anti-inflammatory drug that fails to prevent radiological progression of RA, suppressed the progression of disease and induced joint protection in acute CIA. In contrast, when used in chronic CIA, indomethacin slowed clinical progression but failed to protect against joint destruction [27]. Thus, chronic CIA is a useful model for testing potential therapies for RA. Moreover, the extensive vasculature network in this model [27] provides an ideal platform for testing antiangiogenic agents. To determine whether the efficacy of PPI-2458 in acute CIA translated also to a chronic model of arthritis, which would more closely mimic human RA, we investigated its effect in chronic CIA. In this model, PPI-2458 consistently reduced disease severity and joint destruction.

To evaluate further the mechanism of action of PPI-2458, the effects of the MetAP-2 inhibitors on endothelial proliferation and tubule formation induced by VEGF and FGF-2 were investigated. VEGF and FGF-2 are expressed in human RA and increase endothelial angiogenesis [6,8,43,44]. The *in vivo *efficacy of PPI-2458 in CIA was paralleled by significant inhibition of *in vitro *angiogenesis, which was observed as reduced endothelial proliferation and tubule formation induced both by VEGF and FGF-2. However, the anti-angiogenic effect of PPI-2458 was not accompanied by a global inhibition of endothelial cell responses, because release of cytokines and chemokines by confluent HUVEC exposed to IL-1β, TNF-α, or LPS was not affected at the same doses of PPI-2458. Furthermore, PPI-2458 did not affect total RA synovial cell responses. Release of IL-6 and VEGF from RA fibroblasts has been shown to be unaffected by PPI-2458 [28]. However, in addition to the fibroblasts, many other cell types are present in RA synovium, and these cells are also sources of proinflammatory cytokines, particularly TNF-α in the case of macrophages. The heterogeneous total RA synovial membrane cell culture system was originally developed to examine the effectiveness of TNF-α blockade in RA [45], and it has since been extensively used to study synovial cell responses, including expression of angiogenic factors such as VEGF [6,46-49]. In this model, release of chemokines (IL-8, MCP-1, IP-10 and MIP-1α), cytokines (IL-6 and TNF-α) and growth factors (VEGF) was unaffected by PPI-2458.

We have shown that long-term administration of PPI-2458 reduces chronic relapsing CIA, a model that has previously been shown to mimic human RA, and to abolish endothelial cell angiogenic responses to key mediators, namely VEGF and FGF-2. Taken together, these results highlight that MetAP-2 is a good candidate therapeutic intervention in RA. Although we have not unequivocally demonstrated that angiogenesis is inhibited by PPI-2458 *in vivo*, the *in vitro *findings suggest that inhibition of MetAP-2 might translate to reduced blood vessel formation in arthritic synovium. However, more detailed analysis of synovial vascularity would be required to substantiate this hypothesis.

## Conclusion

The chronic model of arthritis closely mimics that of RA and hence is a potentially a good predictor of clinical efficacy. The response in chronic CIA to MetAP-2 inhibition indicates that PPI-2458 has therapeutic potential for future use in RA.

## Abbreviations

CIA = collagen-induced arthritis; ELISA = enzyme-linked immunosorbent assay; FCS = foetal calf serum; FGF = fibroblast growth factor; HUVEC = human umbilical vein endothelial cell; IL = interleukin; IP = interferon-γ-inducible protein; LPS = lipopolysaccharide; MCP = monocyte chemoattractant protein; MetAP = methionine aminopeptidase; MIP = macrophage inflammatory protein; RA = rheumatoid arthritis; sTNF-R = soluble tumour necrosis factor receptor; TNF = tumour necrosis factor; VEGF = vascular endothelial growth factor.

## Competing interests

Dr Michael Binks and Dr Rajneesh Malhotra are employees of GlaxoSmithKline and declare competing financial interests. The other authors declare that they have no competing interests.

## Authors' contributions

EP was the principal investigator for this study and oversaw the data analysis and drafting of the manuscript. RM and MB assisted in the study design and co-ordination. JB designed and carried out the *in vivo *CIA studies. LM designed and carried out the *in vitro *endothelial studies. DE processed samples for histological analyses and assisted in their analysis. All authors have read and approved the final manuscript.
